# Corticotropin Releasing Factor Binding Protein as a Novel Target to Restore Brain Homeostasis: Lessons Learned From Alcohol Use Disorder Research

**DOI:** 10.3389/fnbeh.2021.786855

**Published:** 2021-11-29

**Authors:** Dallece E. Curley, Ashley E. Webb, Douglas J. Sheffler, Carolina L. Haass-Koffler

**Affiliations:** ^1^Center for Alcohol and Addiction Studies, Brown University, Providence, RI, United States; ^2^Neuroscience Graduate Program, Department of Neuroscience, Brown University, Providence, RI, United States; ^3^Department of Molecular Biology, Cell Biology, and Biochemistry, Brown University, Providence, RI, United States; ^4^Carney Institute for Brain Science, Brown University, Providence, RI, United States; ^5^Cell and Molecular Biology of Cancer Program, Sanford Burnham Prebys Medical Discovery Institute, La Jolla, CA, United States; ^6^Conrad Prebys Center for Chemical Genomics, Sanford Burnham Prebys Medical Discovery Institute, La Jolla, CA, United States; ^7^Department of Psychiatry and Human Behavior, Warren Alpert Medical School, Brown University, Providence, RI, United States; ^8^Department of Behavioral and Social Sciences, School of Public Health, Brown University, Providence, RI, United States

**Keywords:** CRFBP, AUD, stress, neurodegeneration, HPA axis, aging

## Abstract

Stress is well-known to contribute to the development of many psychiatric illnesses including alcohol and substance use disorder (AUD and SUD). The deleterious effects of stress have also been implicated in the acceleration of biological age, and age-related neurodegenerative disease. The physio-pathology of stress is regulated by the corticotropin-releasing factor (CRF) system, the upstream component of the hypothalamic-pituitary-adrenal (HPA) axis. Extensive literature has shown that dysregulation of the CRF neuroendocrine system contributes to escalation of alcohol consumption and, similarly, chronic alcohol consumption contributes to disruption of the stress system. The CRF system also represents the central switchboard for regulating homeostasis, and more recent studies have found that stress and aberrations in the CRF pathway are implicated in accelerated aging and age-related neurodegenerative disease. Corticotropin releasing factor binding protein (CRFBP) is a secreted glycoprotein distributed in peripheral tissues and in specific brain regions. It neutralizes the effects of CRF by sequestering free CRF, but may also possess excitatory function by interacting with CRF receptors. CRFBP’s dual role in influencing CRF bioavailability and CRF receptor signaling has been shown to have a major part in the HPA axis response. Therefore, CRFBP may represent a valuable target to treat stress-related illness, including: development of novel medications to treat AUD and restore homeostasis in the aging brain. This narrative review focuses on molecular mechanisms related to the role of CRFBP in the progression of addictive and psychiatric disorders, biological aging, and age-related neurodegenerative disease. We provide an overview of recent studies investigating modulation of this pathway as a potential therapeutic target for AUD and age-related neurodegenerative disease.

## Introduction

The deleterious effect of stress, both as an acute insult or chronic exposure, has been linked to many pathophysiological changes in the human body. The brain is the central hub for the stress response, which controls both physiological and behavioral mechanisms that regulate health outcomes. Therefore, stress is considered a major contributor to the initiation and development of a variety of psychiatric disorders, including alcohol and substance use (AUD and SUD) (Koob, 2008; Sinha, 2008), anxiety (Pêgo et al., 2010; Nolte et al., 2011), and depression (Hammen, 2005). Further, stress has been implicated in the acceleration of biological aging (Epel et al., 2004; Wolf and Morrison, 2017; Yegorov et al., 2020) and in the progression of neurodegenerative diseases (Hemmerle et al., 2012; Djamshidian and Lees, 2014). Chronic psychological stress has been found to mechanistically contribute to acceleration of aging through cellular senescence (Yegorov et al., 2020) and stimulation of the pro-inflammatory response (Rea et al., 2018; Yegorov et al., 2020), which in turn may result in massive death of neurons (Bachis et al., 2008; de Pablos et al., 2014). Therefore, stress-induced acceleration of brain aging can initiate pathological processes that can lead to early onset and progression of dementia (Peavy et al., 2012), Parkinson’s (Hemmerle et al., 2012), and Alzheimer’s (Swaab et al., 2002; Swerdlow, 2011; Mosher and Wyss-Coray, 2014) diseases. Despite the clear evidence of the role of stress on the development of many brain diseases and in accelerating the process of biological aging, currently there are no medications that target the stress system. Furthermore, there are no therapeutic interventions designed to re-establish the homeostatic imbalance produced by acute or chronic stress insults.

In order to adapt to stress, the hypothalamic-pituitary-adrenal (HPA) axis initiates a cascade of neuroendocrine processes characterized by release of corticotropin releasing factor (CRF) from the paraventricular nucleus of the hypothalamus (PVN), which binds to two G protein-coupled receptors (GPCRs) CRF_1_ and CRF_2_ (Bale and Vale, 2004). After almost two decades of intense research in developing pharmacophores that target the stress system at the central nervous system (CNS) level, clinical studies utilizing CRF_1_ antagonists failed to translate to the clinical setting, and lacked efficacy in trials for individuals with AUD (Binneman et al., 2008), major depression and anxiety disorders (Spierling and Zorrilla, 2017).

**FIGURE 1:**
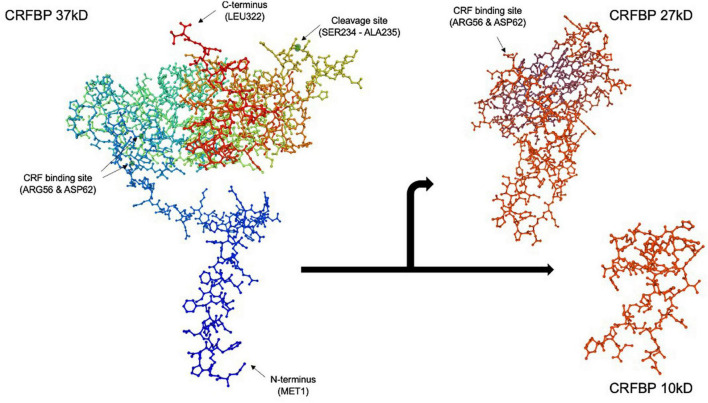
Corticotropin releasing factor binding protein (CRFBP) 37 kD model by SWISS-MODEL protein structure homology. CRFBP is composed of 322 amino acid residues, spanning from the N-terminus at methionine 1 (MET1) to the C-terminus at leucine 322 (LEU322). Spontaneous, proteolytic cleavage occurs between amino acid residues serine 234 (SER234) and alanine 235 (ALA235), resulting in formation of the 27 kD N-terminal and 10 kD C-terminal fragments. The high affinity, CRF selective binding sites located at arginine 56 (ARG56) and aspartic acid 62 (ASP62) are retained by the 27 kD fragment. The 27 kD fragment, which acts as a scavenger of excess CRF, is believed to be responsible for the inhibitory role of CRFBP. Conversely, studies indicate the 10 kD fragment may possess excitatory functions through its selective interaction with CRFR_2_. The full 322 amino acid CRFBP (37 kD), N-terminal fragment (27 kD) (reduced amino acid sequence 62-212), and C-terminal fragment (10 kD) (reduced amino acid sequence 45–85) figures were computed using SWISS-MODEL (Guex et al., 2009; Bienert et al., 2017; Waterhouse et al., 2018).

Determining the biochemical perturbations that disturb brain homeostasis at the cellular, tissue and organ level is critical to develop therapies for disorders that are mediated by the stress response. Recently, translational efforts in the AUD field have contributed to evaluating the role of CRF binding protein (CRFBP) as a potential target to modulate the stress system (Haass-Koffler, 2018; Figure 1). In preclinical settings, research has utilized *CRHBP* deficient (–/–) mice to examine phenotypic consequences of stress and the impact on stress-related behaviors (Ketchesin et al., 2017). In humans, genetic variations in CRFBP have been shown to predict antidepressant outcomes in randomized controlled trials (RCTs) (O’Connell et al., 2018). Interestingly, sequestration of CRF by CRFBP has been proposed as a target for Alzheimer’s disease, due to its potential to restore normal stress functioning and improve symptoms of brain aging (Vandael and Gounko, 2019).

In this review, we will examine the role of CRFBP as a target to restore imbalances in brain homeostasis with the intent to treat brain illnesses. We will first review the role of CRFBP in the HPA axis stress response, briefly summarizing literature characterizing its actions within the central nervous system (CNS). Then, we will examine the research efforts in the AUD field, as a potential target for inhibiting stress-induced alcohol consumption. Finally, we will review the role of CRFBP as a potential target for treating other SUD, anxiety, depression, and some neurodegenerative disorders.

## Mechanistic Role of Corticotropin Releasing Factor Binding Protein in the Stress Response

After a stress stimulus, the initial secretion of CRF from the PVN stimulates the anterior pituitary gland to release adrenocorticotropic hormone (ACTH) in the systemic circulation (Raff, 1993). The release of ACTH then triggers the adrenal cortex to release glucocorticoid (GC) hormones, which exert inhibitory feedback on the HPA axis to regulate its function (Keller-Wood and Dallman, 1984; Tasker and Herman, 2011). Thus, the HPA axis, from central factor activation, to mobilization of hormones into the peripheral pathways, provides multiple targets that have been the focus of many research studies over the last couple of decades (Pomara et al., 2003; Du and Pang, 2015; DeMorrow, 2018; Canet et al., 2019). Preclinical studies have strongly supported CRF as a key mediator of neuroendocrine and behavioral responses to stress (Gray, 1993; Heinrichs et al., 1995; Bale and Vale, 2004), particularly in AUD and SUD (Koob and Kreek, 2007; Koob and Le Moal, 2008; Mukhara et al., 2018). While research has primarily focused on the actions of CRF and GCs, CRFBP has been suggested as a promising novel target for the stress component of AUD and stress-related illnesses. Since its discovery (Linton et al., 1988) and isolation from the human plasma (Behan et al., 1989), CRFBP has been proposed as valuable stress scavenger due to its ability to prevent the excessive stress response through limiting and regulating CRF bioavailability in the HPA axis (Potter et al., 1991, 1992; Sutton et al., 1995).

Corticotropin releasing factor binding protein is a 37 kDA soluble, secreted glycoprotein that is highly conserved from invertebrates to humans (Haass-Koffler and Bartlett, 2012; Ketchesin et al., 2017). In humans and non-human primates, CRFBP is dispersed in discrete brain regions as well as peripherally in the liver and placenta (Potter et al., 1991, 1992; Petraglia et al., 1993; Kemp et al., 1998; Slater et al., 2016) where it regulates the function and bioavailability of CRF. In other mammals including rodents, CRFBP is not expressed in peripheral tissue, which has limited research on CRFBP’s full mechanistic role in the stress system in preclinical studies (Potter et al., 1991, 1992). CRFBP is also an extremely versatile protein that may interact, not only through sequestration of excess CRF, but by interacting with the CRF receptors in an allosteric manner. Interestingly, a potential approach to treat stress-related disorders would be by utilizing CNS-penetrant pharmacophores that may modulate the affinity of CRF for the CRF receptors, rather than blocking neural connectivities by binding to orthosteric sites (Haass-Koffler, 2018). The approach of disrupting a three-way interaction between ligand, receptor, and macromolecule, further supports the involvement of additional peptides that may influence the pathophysiology of the CRF system (Spierling and Zorrilla, 2017).

Corticotropin releasing factor binding protein is composed of 322 amino acid residues and approximately 70% undergoes proteolytic cleavage in the brain (Behan et al., 1995a; Figure 1). Spontaneous cleavage between amino acid residues serine 234 and alanine 235 yields an N-terminal fragment CRFBP (27 kD) and a C-terminal fragment CRFBP (10 kD), through a mechanism that has yet to be elucidated (Woods et al., 1999). The 27 kD fragment retains the CRF selective and high affinity binding site at arginine 56 and aspartic acid 62 (IC_50_ = 0.5 nM) (Woods et al., 1999). The biological role of the 10 kD fragment remains unknown.

*In vitro* studies indicate that CRFBP (27 kD) is responsible for the inactivation of “free” CRF, while CRFBP (10 kD) may have an excitatory role interacting selectively with CRFR_2_ (Haass-Koffler et al., 2016). Interestingly, during stress induction, it has been hypothesized that CRFBP (27 kD) is unable to sequester the free CRF, and the CRF in excess with CRFBP (10 kD) allosterically potentiates CRFR_2_ signaling in a three-way interaction (Haass-Koffler et al., 2016). To support this hypothesis, examination of the interaction between CRFBP and CRF_2_ in cultured mesencephalic neurons demonstrated that CRFBP interacts with intracellular CRF_2_ to increase presence of the receptor on the cell surface (Haass-Koffler et al., 2012), acting as a GPCR escort protein (Slater et al., 2016).

Electrophysiological studies showed that CRF potentiation of NMDAR (*N*-methyl-D-aspartate receptor)-mediated synaptic transmission in dopamine neurons of the ventral tegmental area (VTA) involves CRF_2_ and requires CRFBP for potentiation (Ungless et al., 2003). In the VTA, NMDAR activity is believed to mediate stress-induced activation of the dopamine system (Overton and Clark, 1997), which is associated with alterations in behaviors underlying SUDs (Shaham et al., 2000). As such, this provides a plausible mechanism by which modulation of CRFBP can mediate stress-induced changes contributing to SUDs. Further, double *in situ* hybridization of CRFBP in the VTA showed that CRFBP mRNA is expressed within a subset of dopaminergic neurons, but not in the neighboring substantia nigra pars compacta (SNC) (Wang and Morales, 2008). In summary, findings of *in vitro* studies indicate that complex mechanistic interactions between CRFBP, CRF, and CRF receptors play an important role in CRFBP’s ability to regulate neurotransmission. The *ex vivo* studies showed that this effect may be relevant in brain regions such the VTA that have been implicated with AUD and SUD, and possibly also in neurodegenerative disorders (Ungless et al., 2003; Haass-Koffler, 2018). Of note, in mouse models of AD, degeneration of VTA dopaminergic neurons at pre-plaque stages contributes to memory deficits and dysfunction of reward processing (Nobili et al., 2017).

The development of the *CRHBP* deficient (–/–) mice allowed researchers to evaluate the impact of CRFBP on different bio-behavioral effects (Karolyi et al., 1999; Ketchesin et al., 2017). Early studies showed that *CRHBP* deficiency (–/–) in mice contributed to increased stress and anxiety-like behaviors compared to wildtype littermates (Karolyi et al., 1999). Interestingly, the same study found that *CRHBP* deficient (–/–) mice did not alter HPA axis functioning, as there were no significant differences in basal levels of corticosterone or ACTH compared to wildtype littermates (Karolyi et al., 1999). An additional transgenic mouse model which overexpressed CRFBP in the anterior pituitary gland, showed an increased motor activity, and a trend in decreased anxiety compared to wildtype littermates (Burrows et al., 1998). Similar to *CRHBP* deficient (–/–) mice, transgenic mice overexpressing pituitary CRFBP did not show any differences in basal levels of corticosterone or ACTH compared to wildtype littermates (Burrows et al., 1998; Lovejoy et al., 1998). However, significant elevation of both CRF and vasopressin in the PVN of the transgenic CRFBP overexpressing mice compared to littermates indicates that compensatory mechanisms occur in response to CRFBP overexpression in order to maintain homeostasis (Burrows et al., 1998). Further, when the HPA axis of transgenic mice overexpressing CRFBP was stimulated through injections of lipopolysaccharide, ACTH significantly decreased, indicating that higher levels of circulating CRFBP are needed to alter HPA axis function (Lovejoy et al., 1998).

Recently, it was reported sex-dependent mechanisms underlying the expression of CRFBP in rodents. A recent study in C57BL/6J mice found that hypothalamic CRFBP was upregulated in 12-month-old male mice compared to females, and conversely downregulated in female mice at 18 months (Locci et al., 2021). Similar sex-dependent changes were observed in CRFBP expression in the amygdala at different time points, in addition to sex-specific differences in acute restraint stress-induced alterations in CRFBP expression (Locci et al., 2021). This finding aligns with previous studies, which found that female C57BL/6J mice had greater CRFBP expression in the pituitary compared to male mice (Speert et al., 2002; Stinnett et al., 2015). Similarly, female adult Long Evans rats exhibit greater CRFBP expression in the medial septum compared to male rats, a brain region important for cognitive functioning (Wiersielis et al., 2019).

## Corticotropin Releasing Factor Binding Protein and Alcohol Use Disorder

Alcohol use disorder is characterized by habitual, excessive consumption of alcohol, leading to negative physical, emotional, and social consequences. Alcohol is unique from other drugs as it binds many different molecular targets and alters various neurotransmitter systems in the body, including endogenous opioids, γ-Aminobutyric acid (GABA), serotonin, glutamate, and dopamine (Lovinger, 1997; Trantham-Davidson and Chandler, 2015; Abrahao et al., 2017). The alcohol research field has increased its focus on the stress system, which has been identified as a major contributor to the development, progression, and reinforcement of behaviors underlying AUD (Brady and Sonne, 1999; Sarnyai et al., 2001). Importantly, researchers have found that both stress and alcohol activate the mesolimbic reward pathway, and regulate neurotransmitter activity in limbic brain regions, further supporting interest in mechanistic targets within the stress system (Haass-Koffler and Bartlett, 2012; Mukhara et al., 2018). Early studies focused on directly targeting CRF to mediate stress-induced alcohol-related behaviors (Rodriguez and Coveñas, 2017; Schreiber and Gilpin, 2018). However, more recently, researchers have examined the regulatory role of CRFBP in the CRF stress pathway as an indirect target to alter CRF levels and HPA axis activity (Haass-Koffler et al., 2016; Haass-Koffler, 2018). This interest has been further supported by findings of CRFBP expression in discrete subpopulations of dopaminergic and GABAergic neurons in the VTA (Wang and Morales, 2008), a brain pathway which is known to be important in regulating reward, reinforcement, and cognition in AUD (You et al., 2018).

Intra-VTA microinjections of the CRFBP antagonist, CRF_6–33_, in C57BL/6J mice dose-dependently reduced ethanol consumption, suggesting that CRFBP antagonism in the VTA may contribute to increased alcohol consumption (Albrechet-Souza et al., 2015). Those data corroborate the previous electrophysiology studies that tested CRFBP’s role in the VTA (Ungless et al., 2003), demonstrating that CRF requires CRFBP to potentiate NMDA receptors *via* CRF_2_ (Ungless et al., 2003). A recent study that evaluated the colocalization of CRFBP and CRF2R in cultured rat mesencephalic neurons supports this early data and demonstrated that CRFBP facilitates the presence of CRF_2_ on the cell surface (Slater et al., 2016).

The central nucleus of the amygdala (CeA) is an important limbic brain region relevant to AUD, as it has been identified as a hub for negative emotional circuitry and alcohol reinstatement (Roberto et al., 2012; Simms et al., 2012). However, studies investigating the role of CRFBP in the CeA have cast inconsistent results. Early findings in rats examining expression of CRFBP mRNA expression found that CRFBP is localized in key limbic regions, including the CeA (Potter et al., 1992). Pharmacological approaches, using microinjections of the CRFBP antagonist, CRF_6–33_, into the CeA of C57BL/6J mice were found to have no effect on ethanol intake (Albrechet-Souza et al., 2015). Downregulating CRFBP in the CeA using lentivirus vectors and *s*equences encoding small hairpin RNAs (shRNAs) targeting the *CRHBP* gene, decreased ethanol consumption in rats (Haass-Koffler et al., 2016). The downregulation of *CRHBP* in the CeA, however, was unable to block the subsequent challenge by yohimbine-induced ethanol self-administration (Haass-Koffler et al., 2016). This effect was reflected in a functional MRI experiment which showed that in the CeA there was a lower hemodynamic activation in *CRHBP* shRNA rats at baseline compared to controls (*Scr* shRNA) and after yohimbine administration there was an increase in hemodynamic activation in the *Scr* shRNA rats only. Those results were also corroborated by the fact that in other brain regions known to be activated after yohimbine administration (e.g., caudate putamen), there was hemodynamic activation in both *CRHBP* and *Scr* shRNA rats (Haass-Koffler et al., 2016). Altogether, these findings suggest that downregulation of CRFBP in the CeA alone may not be sufficient to mediate stress-induced alcohol consumption, particularly during noradrenergic activation (Haass-Koffler et al., 2016, 2018).

In preclinical alcohol behavioral studies, the impact of binge drinking on CRFBP expression across relevant brain regions was examined in C57BL/6J mice using repeated drinking-in-the-dark (DID) cycles. *In situ* hybridization findings indicated that repetitive binge drinking significantly decreases CRFBP mRNA expression in the prelimbic (PL) and infralimbic medial prefrontal cortex (mPFC) after three cycles of DID, and in the PL mPFC after six cycles (Ketchesin et al., 2016). Notably, there were no observed changes in CRF or CRF_1_ mRNA in the mPFC, VTA, bed nucleus of the stria terminalis, or amygdala (Ketchesin et al., 2016). The DID paradigm was also paired to the CRFBP transgenic mouse model, showing that alcohol consumption was significantly higher in *CRHBP* deficient (–/–) mice compared to their wildtype littermates. This effect was statistically significant after repeated DID sessions with a strong distinction after the sixth DID exposure (Haass-Koffler et al., 2016). Interestingly, studies examining acute restraint stress observed increased CRFBP mRNA expression in the rodent pituitary (McClennen et al., 1998; Stinnett et al., 2015) and amygdalar regions (Lombardo et al., 2001; Herringa et al., 2004; Roseboom et al., 2007); however, chronic restraint was associated with decreases in amygdalar CRFBP mRNA expression (Pisarska et al., 2000). These findings highlight different mechanisms through which acute and chronic stress regulate central expression of CRFBP.

Overall, these findings represent promising preliminary evidence to support further preclinical investigation of CRFBP as a target for alcohol-related behaviors. Specifically, future studies should focus on examining robust models of behavioral and pharmacologically-induced stress, to further elucidate the mechanistic role of CRFBP in AUD, and its ability to mediate dysregulation of the stress response within preclinical models of AUD.

As CRFBP is still a novel therapeutic target undergoing investigation, clinically relevant research examining CRFBP in AUD remains limited to human genetic studies. Translation of *in vitro* findings to human genetics research has led to preliminary evaluation of several single nucleotide polymorphisms (SNPs) of the *CRHBP* gene. Studies that evaluated SNPs that reside in the *CRHBP* (10 kD) gene have shown to be associated with stress-induced craving in non-treatment-seeking individuals who drink heavily (Ray, 2011; Table 1). A study examining individuals with AUD found associations between three SNPs (*rs10062367, rs7718461*, and *rs10055255*) and alcohol and anxiety-related phenotypes (Haass-Koffler et al., 2016; Table 1). One of the SNPs (*rs10062367*) was associated with increased risk of these phenotypes in African and European individuals, while the other two SNPs (*rs7718461* and *rs10055255*) were found to be linked to decreased anxiety-related phenotypes in Europeans (Haass-Koffler et al., 2016; Table 1).

**TABLE 1:** Association between single nucleotide polymorphisms (SNPs) of the *CRHBP* gene, psychiatric and neurodegenerative disorders.

*CRHBP* SNP	Allele	*N*	Ancestry	Diagnosis	Outcome	Risk	References
*rs10062367*	A	476	Full	AUD	Alcohol consumption	↑	Haass-Koffler et al., 2016
		206	African			↑	
		86	European		Anxiety symptoms	↑	
*rs7718461*	A	220	European		Anxiety symptoms	↓	
*rs1053989*	C	223			Neuroticism	↓	
		221			Anxiety symptoms	↓	
*rs10474485*	–	554	European	AUD	Depressive symptoms	↑	Kertes et al., 2011
*rs1715747*	G				Depressive symptoms	↑	
*rs10055255*	T	41	Full	AUD	Stress-induced negative affect and negative consequences of drinking	↑	Tartter and Ray, 2012
*rs1500*	C	351	NS*	OUD and CUD	Cocaine and benzodiazepine use	↑	Peles et al., 2019
*rs3792738*	A	336	Asian	HUD	Stress symptoms	↓	Su et al., 2018
*rs1875999*	T	177	European	MDD and matched controls	MDD symptoms	↑	Claes et al., 2003
*rs7728378*							
*rs28365143*	G	636	Full	MDD	Depression symptoms	↓	O’Connell et al., 2018
*rs10473984*	T	1953	Full	MDD with anxious depressive symptoms	Treatment response	↓	Binder et al., 2010
		399	African and Hispanic				

CUD, cocaine use disorder; HUD, heroin use disorder; MDD, major depressive disorder; NS*, not specified; OUD, opioid use disorder.

Furthermore, polymorphism in the CRFBP10 kD has been observed in individuals with diagnosis of AUD (Enoch et al., 2008) and suicide risk (Roy et al., 2012). In individuals with AUD, two SNPs (*rs10474485* and *rs1715747*) in the *CRHBP* gene were significantly associated with increased depressive symptoms scores (Kertes et al., 2011; Table 1). A subsequent study found that *CRHBP* genotype (*rs10055255*) moderated the relationship between stress-induced negative affect, and the negative consequences of drinking in young adults who drink heavily (Tartter and Ray, 2012; Table 1). These studies further support the role of CRFBP not only in AUD, but other mental health outcomes.

Although findings linking genetic polymorphisms to stress and AUD are promising for the development of diagnostic markers, prognostic tools and therapeutic development, additional translational research is needed to identify clinically translatable approaches for targeting CRFBP in humans.

## Potential Role of Corticotropin Releasing Factor Binding Protein in Other Stress-Related Psychiatric Disorders

As stress has been well established to play a role in the pathogenesis of various neuropsychiatric disorders, many studies over the years have focused on targeting the CRF system to mediate dysregulation of the HPA axis (Binder and Nemeroff, 2010; Haass-Koffler and Bartlett, 2012). However, despite promising preclinical findings, clinical trials targeting CRF and CRF_1_ have shown limited or negligent success in treating a range of psychiatric conditions, including SUD (Lijffijt et al., 2014), anxiety and depressive, and neurodegenerative disorders (Sanders and Nemeroff, 2016; Spierling and Zorrilla, 2017). Thus, targeting of CRFBP may similarly hold promise for the treatment of the stress-related components of these disorders.

The role of CRFBP in seeking of other drugs has also undergone limited investigation within rodent models. For example, the role of VTA CRFBP in stress-induced reinstatement of cocaine seeking was examined in Long Evans rats (Wang et al., 2007). The study found that perfusing CRF-like agonists (CRF, UCN I, and mouse UCN II) which avidly bind CRFBP, increased glutamate and dopamine levels within the VTA, and also reinstated cocaine seeking (Wang et al., 2007). However, intra-VTA infusions of agents which do not bind CRFBP (stressin I, UCN III, and ovine CRF) did not increase the neurotransmitter levels, nor did they reinstate cocaine seeking (Wang et al., 2007). The finding that CRFBP agonism in the VTA increases drug seeking compliments research in the AUD field, which found that antagonism of CRFBP in the VTA reduces alcohol consumption (Albrechet-Souza et al., 2015).

In individuals with concurrent opioid and cocaine use disorder who underwent methadone maintenance therapy for at least a year, the *CRHBP* SNP (*rs1500* C allele) was linked with cocaine and benzodiazepine consumption (Peles et al., 2019). The *rs1500* C allele was a predictor of cocaine and benzodiazepine 1 year into treatment (Peles et al., 2019; Table 1).

Similarly, associations between the stress pathway and heroin use have been identified through analysis of polymorphisms in *CRHBP* (Su et al., 2018). A study examining nine SNPs in stress-related genes in abstinent individuals with heroin use disorder and healthy controls, found no genotypic differences between these groups, however, multivariate regression analysis identified one *CRHBP* variant (*rs3792738*), as a predictor risk of heroin relapse in patients with chronic stress (Su et al., 2018; Table 1).

Identification of variations in the *CRHBP* gene and stress pathway have been associated with development of several SUDs and relapse susceptibility (Levran et al., 2014, 2018), which holds promise for the development of personalized therapeutic treatments. However, those results are limited to human genetic study targeting individual SNPs. Extensive genome-wide association studies (GWAS) approaches within different SUDs are necessary to further elucidate the mechanistic role of CRFBP in this population.

The link between the CRF system and anxiety and depressive disorders has been well supported in preclinical studies (Arborelius et al., 1999). As CRFBP is found in all CNS CRF-related pathways and the pituitary, research investigating the role of CRFBP specifically in anxiety disorders to support development of pharmacotherapies is of great interest. Early studies in patients with major depressive disorder (MDD) identified several SNPs associated with vulnerability for major depression (Claes et al., 2003; Van Den Eede et al., 2007). In an isolated population of Swedish individuals with recurrent MDD and matched controls, two SNPs (*rs1875999* and *rs7728378*) in the *CRHBP* gene were found to be significantly associated with occurrence of MDD in the patients with the variant compared to control individuals (Claes et al., 2003; Table 1). In a follow up study, an extended sample of Swedish patients and an independent sample of Belgian patients with MDD (and their respective matched controls) were examined to replicate and confirm the original findings (Van Den Eede et al., 2007). Unfortunately, the findings could not be replicated in the independent Belgian population, and the results of the extended Swedish population differed from the original study in that only an overall trend was observed. However, analysis of sex differences identified significant associations of three *CRHBP* SNPs (*rs7728378*, *rs1875999*, and *rs1052967*) with occurrence of MDD in Swedish males (Van Den Eede et al., 2007; Table 1). These observed sex differences in humans are in line with preclinical findings, which indicated sexual dimorphisms exist in the expression of *CRHBP* in the pituitary of mice (Speert et al., 2002).

More recently, researchers have expanded investigation of the *CRHBP* gene to assess the role that genetic variants play in response to antidepressant medications. In individuals with non-psychotic MDD, without comorbid substance or psychiatric conditions, 16 candidate HPA axis SNPs were investigated as predictors of treatment outcomes for three antidepressants (escitalopram, sertraline and venlafaxine-XR) (O’Connell et al., 2018). Only one of the 16 SNPs, (*CRHBP rs28365143*) was found to significantly predict reduction in Hamilton Depression Rating Scale scores (O’Connell et al., 2018; Table 1). Individuals with the *rs28365143* homozygous G allele, reported significantly better outcomes than A allele carriers, notably only for the escitalopram and sertraline antidepressants (both selective serotonin reuptake inhibitor, SSRIs), while no association was found with venlafaxine-XR treatment (serotonin-norepinephrine reuptake inhibitor, SNRIs) (O’Connell et al., 2018; Table 1). There has long been controversy within the field as to whether SSRIs or SNRIs are more effective in treating depressive disorders, as findings have been highly variable (Thase, 2008). Genetic variations in the *CRHBP* gene could provide additional insight into these differential responses, supporting the development of personalized medicine approaches for pharmacological treatments.

This approach can be further informed through investigation of *CRHBP* genetic variations among diverse populations. For example, a study of European American, African American, and Hispanic patients with non-psychotic MDD examined associations of genetic variants in 10 genes that regulate the CRF and arginine vasopressin systems with antidepressant treatment response (citalopram, SSRI) (Binder et al., 2010). Of these 10 genes, only one SNP *rs10473984* within the *CRHBP* locus was significantly associated with treatment outcome (remission and reduction in depressive symptoms) in response to citalopram (Binder et al., 2010). In African Americans and Hispanics, *rs10473984* T allele was associated with worse treatment outcomes compared to European Americans (although population stratification and admixture among ethnic groups present possible confounding variables) (Binder et al., 2010; Table 1). This observation may position ethnic background as an important factor to be considered in CRFBP’s role in antidepressant treatment response.

Another relevant consideration is the high occurrence of comorbid depressive disorder and SUD (Currie et al., 2005; Brière et al., 2014). Investigation of *CRHBP* genetic polymorphisms can provide additional insight on the role that *CRHBP* variants play in genetic risk for comorbid depression and other psychiatric conditions. For example, a study examining the history of depressive symptoms in individuals with AUD analyzed markers in 120 candidate genes relevant to SUD’s and anxiety or depression (Kertes et al., 2011). Of the 1,350 SNPs assessed, only three met or exceeded the significance threshold for association with depressive symptom scores (*CRHBP rs1715747, GABRB1 rs4315750, and OPRM1 rs650245*), although interestingly none of the analyzed markers were associated with AUD symptom scores (Kertes et al., 2011; Table 1).

Overall, these findings support a link between CRFBP and anxiety and depressive disorders. They also highlight the importance of investigating additional population variables, such as ethnic background and sex to inform the development of personalized medicine. Future studies should seek to further utilize preclinical models to examine the mechanistic contributions of CRFBP to the development of anxiety and depressive illnesses.

## Potential Role of Corticotropin Releasing Factor Binding Protein in Other Stress-Related Neurodegenerative Disorders

Chronic stress exposure is believed to accelerate biological aging through mechanistic changes leading to cellular senescence (Yegorov et al., 2020). A review of preclinical and clinical studies suggests that psychological stress increases neuroinflammation, enhancing pro-inflammatory cytokine signaling (Salim, 2016). Increased pro-inflammatory cytokine levels may contribute to oxidative stress and cellular damage through promoting production of reactive oxygen species (ROS) (Salim, 2016), which has been linked to accelerated aging (El Assar et al., 2017). Further, shortening of telomeres, which is considered a hallmark of aging (Jiang et al., 2007), is associated with oxidative stress (Epel et al., 2004). Thus, similar to the proposed role of CRFBP in mediating stress-induced alcohol behaviors, CRFBP may also hold potential for mediating stress-induced acceleration of aging and age-related disorders.

Corticotropin releasing factor binding protein has been suggested as a molecular mechanism linking stress and age-related neurodegenerative disorders, particularly Alzheimer’s disease (AD) (Vandael and Gounko, 2019). Early *in vitro* studies showed that astrocytes express and regulate the release of CRFBP (Behan et al., 1995c; Maciejewski et al., 1996; McClennen and Seasholtz, 1999). Importantly, by contiguously connecting the CNS and regulating neuronal metabolism and maintaining brain homeostasis, astrocytes also have a well-established role in the pathogenesis of neurodegenerative disorders (Li et al., 2011; Ben Haim et al., 2015; Phatnani and Maniatis, 2015). Thus, targeting of astrocytic CRFBP provides a plausible mechanism to mediate the contribution of astrocytes to neurodegenerative diseases (Vandael and Gounko, 2019).

An early study in the *postmortem* brain tissue of individuals with AD and healthy controls found that there was no difference in CRFBP levels measured between groups, except for a significant decrease in CRFBP observed in Brodmann area (BA) 39 (Behan et al., 1997). They did, however, find that both complexed CRF (BA 8, BA 9, BA 22, BA 39, nucleus basalis, and globus pallidus) and free CRF (A 4, BA 39, and caudate) levels were significantly decreased in AD patients compared to controls (Behan et al., 1997). This finding holds importance for targeting of CRFBP to treat AD, as modulating CRFBP could mediate the progression of AD pathology through increasing available CRF. This hypothesis was explored in another study, which again examined *postmortem* tissue of individuals with AD and healthy controls, and further utilized a CRFBP antagonist (CRF_6–33_) to dissociate CRF from CRFBP in a rodent model (Behan et al., 1995b). Immunoassay analysis of standardized brain regions from *postmortem* human tissue similarly showed no difference in CRFBP expression between AD and control brains, however, CRF was significantly decreased in the frontal, parietal, and temporal brain regions of AD patients compared to control (Behan et al., 1995b). In rats, administration of the CRFBP antagonist (CRF_6–33_) was found to significantly improve learning and memory (Morris water maze), without significantly altering stress (elevated plus maze) (Behan et al., 1995b). Another study using transgenic mice, overexpressing CRFBP in the pituitary, observed an increase in CRF and increased motor activity, which could provide a method to mediate HPA axis dysfunction associated with AD (Burrows et al., 1998).

Together, these findings further implicate modulation of CRFBP as a possible target to diminish age-related dysfunction in the brain, accelerated by chronic stress. However, research within preclinical rodent models of AD is necessary to better represent the impact of CRFBP on the AD pathology. Additionally, targeting CRFBP to alter neurotransmission in the dopaminergic neurons of the VTA may hold promise for mediating AD associated alterations in these neurons, which contribute to memory and reward dysfunction (Nobili et al., 2017).

Similarly, the implication of stress in the development of Parkinson’s disease (PD) supports potential targeting of CRFBP to mediate the development and progression of PD (Miller and O’Callaghan, 2008). PD is a progressive neurodegenerative disease characterized by degeneration of dopaminergic neurons in the striatum, leading to motor dysfunction (DeMaagd and Philip, 2015). Targeting CRFBP may reduce progression of PD and symptoms, through altering dopaminergic signaling and function in key brain regions. Particularly relevant are preliminary findings of CRFBP overexpression in the pituitary of mice, which resulted in increased motor activity (Burrows et al., 1998). Future research should examine potential mechanisms by which modulation of CRFBP may alter neuronal survival in neurodegenerative conditions, as well as the ability of CRFBP to mediate the consequences of chronic stress which contribute to age-related disorders.

Studies examining age- and disease-related neurodegeneration are particularly important to AUD research as well, as there is high comorbidity between AUD and dementia, yet the elderly remain largely overlooked within AUD research (Caputo et al., 2012).

## Conclusion

Dysregulation of the CRF stress system is highly associated with psychiatric and neurodegenerative disorders. Over the years, various peptides within the CRF system have been investigated as potential targets for treatment, however, have shown limited efficacy in clinical translation. CRFBP is a novel target to mediate the contribution of stress related dysfunction to these conditions. Although a majority of the research on CRFBP within disorders has been focused on AUD, preliminary findings are also relevant to other stress-related psychiatric conditions and age-related neurodegenerative diseases. Further, there is a common occurrence of comorbidity between stress-related disorders and psychiatric conditions, which implicates CRFBP as a therapeutic target to treat the common mechanisms underlying many illnesses (Brady et al., 2000; McCauley et al., 2012; Flory and Yehuda, 2015). Given the promising preliminary findings, further preclinical and clinical research elucidating the role of CRFBP in stress- and alcohol-related behaviors underlying AUD are necessary to provide support for CRFBP as a pharmacological target to treat AUD. This review provides an overview of CRFBP in other stress-related psychiatric disorders, including SUDs, anxiety and depressive conditions, and age-related neurodegenerative disorders. However, the reviewed literature highlights several knowledge gaps pertaining to our understanding of CRFBP. First, findings in discrete brain regions indicate that CRFBP’s actions within the CNS may be both inhibitory and excitatory, requiring further examination to elucidate brain region-specific mechanisms which alter functioning. Additionally, in-depth investigation of CRFBP within preclinical models of SUDs and stress-related psychiatric and neurodegenerative disorders is required to better understand the role of CRFBP in mediating cognitive and memory deficits. Furthermore, there is a dearth of literature critically examining the sex-dependent mechanisms of CRFBP. In order to better inform personalized medicine approaches, future studies on CRFBP should include examination of sex as a biological variable, as well as consider the relevance of ethnic background and genetic polymorphisms as factors that may alter susceptibility to stress-related disorders. Advancements within the AUD research field relevant to this neuropeptide have the potential to inform further understanding and treatment for many conditions that are contributed to by chronic stress pathology.

## Author Contributions

DC and CH-K wrote the manuscript. CH-K supervised the project. AW and DS contributed to writing the manuscript. All authors approved the final version of the manuscript and agreed to be accountable for the content of the work.

## Conflict of Interest

The authors declare that the research was conducted in the absence of any commercial or financial relationships that could be construed as a potential conflict of interest.

## Publisher’s Note

All claims expressed in this article are solely those of the authors and do not necessarily represent those of their affiliated organizations, or those of the publisher, the editors and the reviewers. Any product that may be evaluated in this article, or claim that may be made by its manufacturer, is not guaranteed or endorsed by the publisher.

## References

[B1] Abrahao K. P. Salinas A. G. Lovinger D. M. (2017). Alcohol and the brain: neuronal molecular targets, synapses, and circuits. *Neuron* 96 1223–1238. 29268093 10.1016/j.neuron.2017.10.032 PMC6566861

[B2] Albrechet-Souza L. Hwa L. S. Han X. Zhang E. Y. DeBold J. F. Miczek K. A. (2015). Corticotropin releasing factor binding protein and CRF2 receptors in the ventral tegmental area: modulation of ethanol binge drinking in C57BL/6J mice. *Alcohol. Clin. Exp. Res.* 39 1609–1618. 10.1111/acer.12825 26247973 PMC4558332

[B3] Arborelius L. Owens M. J. Plotsky P. M. Nemeroff C. B. (1999). The role of corticotropin-releasing factor in depression and anxiety disorders. *J. Endocrinol.* 160 1–12. 10.1677/joe.0.1600001 9854171

[B4] Bachis A. Cruz M. I. Nosheny R. L. Mocchetti I. (2008). Chronic unpredictable stress promotes neuronal apoptosis in the cerebral cortex. *Neurosci. Lett.* 442 104–108. 10.1016/j.neulet.2008.06.081 18621098 PMC2543936

[B5] Bale T. L. Vale W. W. (2004). CRF and CRF receptors: role in stress responsivity and other behaviors. *Annu. Rev. Pharmacol. Toxicol.* 44 525–557. 10.1146/annurev.pharmtox.44.101802.121410 14744257

[B6] Behan D. P. De Souza E. B. Lowry P. J. Potter E. Sawchenko P. Vale W. W. (1995a). Corticotropin releasing factor (CRF) binding protein: a novel regulator of CRF and related peptides. *Front. Neuroendocrinol.* 16:362–382. 10.1006/frne.1995.1013 8557170

[B7] Behan D. P. Heinrichs S. C. Troncoso J. C. Liu X. J. Kawas C. H. Ling N. (1995b). Displacement of corticotropin releasing factor from its binding protein as a possible treatment for Alzheimer’s disease. *Nature* 378 284–287. 10.1038/378284a0 7477348

[B8] Behan D. P. Maciejewski D. Chalmers D. De Souza E. B. (1995c). Corticotropin releasing factor binding protein (CRF-BP) is expressed in neuronal and astrocytic cells. *Brain Res.* 698 259–264. 10.1016/0006-8993(95)01014-m 8581494

[B9] Behan D. P. Khongsaly O. Owens M. J. Chung H. D. Nemeroff C. B. De Souza E. B. (1997). Corticotropin-releasing factor (CRF), CRF-binding protein (CRF-BP), and CRF/CRF-BP complex in Alzheimer’s disease and control postmortem human brain. *J. Neurochem.* 68 2053–2060. 10.1046/j.1471-4159.1997.68052053.x 9109532

[B10] Behan D. P. Linton E. A. Lowry P. J. (1989). Isolation of the human plasma corticotrophin-releasing factor-binding protein. *J. Endocrinol.* 122 23–31. 10.1677/joe.0.1220023 2549150

[B11] Ben Haim L. Carrillo-de Sauvage M. A. Ceyzériat K. Escartin C. (2015). Elusive roles for reactive astrocytes in neurodegenerative diseases. *Front. Cell. Neurosci.* 9:278. 10.3389/fncel.2015.00278 26283915 PMC4522610

[B12] Bienert S. Waterhouse A. de Beer T. A. Tauriello G. Studer G. Bordoli L. (2017). The SWISS-MODEL repository-new features and functionality. *Nucleic Acids Res.* 45 D313–D319. 10.1093/nar/gkw1132 27899672 PMC5210589

[B13] Binder E. B. Nemeroff C. B. (2010). The CRF system, stress, depression and anxiety-insights from human genetic studies. *Mol. Psychiatry* 15 574–588. 10.1038/mp.2009.141 20010888 PMC3666571

[B14] Binder E. B. Owens M. J. Liu W. Deveau T. C. Rush A. J. Trivedi M. H. (2010). Association of polymorphisms in genes regulating the corticotropin-releasing factor system with antidepressant treatment response. *Arch. Gen. Psychiatry* 67 369–379. 10.1001/archgenpsychiatry.2010.18 20368512

[B15] Binneman B. Feltner D. Kolluri S. Shi Y. Qiu R. Stiger T. (2008). A 6-week randomized, placebo-controlled trial of CP-316,311 (a selective CRH1 antagonist) in the treatment of major depression. *Am. J. Psychiatry* 165 617–620. 10.1176/appi.ajp.2008.07071199 18413705

[B16] Brady K. T. Sonne S. C. (1999). The role of stress in alcohol use, alcoholism treatment, and relapse. *Alcohol. Res. Health* 23 263–271. 10890823 PMC6760383

[B17] Brady K. T. Killeen T. K. Brewerton T. Lucerini S. (2000). Comorbidity of psychiatric disorders and posttraumatic stress disorder. *J. Clin. Psychiatry* 61(Suppl. 7) 22–32. 10795606

[B18] Brière F. N. Rohde P. Seeley J. R. Klein D. Lewinsohn P. M. (2014). Comorbidity between major depression and alcohol use disorder from adolescence to adulthood. *Compr. Psychiatry* 55 526–533. 10.1016/j.comppsych.2013.10.007 24246605 PMC4131538

[B19] Burrows H. L. Nakajima M. Lesh J. S. Goosens K. A. Samuelson L. C. Inui A. (1998). Excess corticotropin releasing hormone-binding protein in the hypothalamic-pituitary-adrenal axis in transgenic mice. *J. Clin. Invest.* 101 1439–1447. 10.1172/JCI1963 9525987 PMC508722

[B20] Canet G. Hernandez C. Zussy C. Chevallier N. Desrumaux C. Givalois L. (2019). Is AD a stress-related disorder? Focus on the HPA axis and its promising therapeutic targets. *Front. Aging Neurosci.* 11:269. 10.3389/fnagi.2019.00269 31611783 PMC6776918

[B21] Caputo F. Vignoli T. Leggio L. Addolorato G. Zoli G. Bernardi M. (2012). Alcohol use disorders in the elderly: a brief overview from epidemiology to treatment options. *Exp. Gerontol.* 47 411–416. 10.1016/j.exger.2012.03.019 22575256 PMC4999313

[B22] Claes S. Villafuerte S. Forsgren T. Sluijs S. Del-Favero J. Adolfsson R. (2003). The corticotropin-releasing hormone binding protein is associated with major depression in a population from Northern Sweden. *Biol. Psychiatry* 54 867–872. 10.1016/s0006-3223(03)00425-6 14573312

[B23] Currie S. R. Patten S. B. Williams J. V. Wang J. Beck C. A. El-Guebaly N. (2005). Comorbidity of major depression with substance use disorders. *Can. J. Psychiatry* 50 660–666. 16276858 10.1177/070674370505001013

[B24] de Pablos R. M. Herrera A. J. Espinosa-Oliva A. M. Sarmiento M. Muñoz M. F. Machado A. (2014). Chronic stress enhances microglia activation and exacerbates death of nigral dopaminergic neurons under conditions of inflammation. *J. Neuroinflammation* 11:34. 10.1186/1742-2094-11-34 24565378 PMC3941799

[B25] DeMaagd G. Philip A. (2015). Parkinson’s disease and its management: part 1: disease entity, risk factors, pathophysiology, clinical presentation, and diagnosis. *P T* 40 504–532. 26236139 PMC4517533

[B26] DeMorrow S. (2018). Role of the hypothalamic-pituitary-adrenal axis in health and disease. *Int. J. Mol. Sci.* 19:986. 10.3390/ijms19040986 29587417 PMC5979578

[B27] Djamshidian A. Lees A. J. (2014). Can stress trigger Parkinson’s disease? *J. Neurol. Neurosurg. Psychiatry* 85 878–881. 10.1136/jnnp-2013-305911 24259593

[B28] Du X. Pang T. Y. (2015). Is dysregulation of the HPA-axis a core pathophysiology mediating co-morbid depression in neurodegenerative diseases? *Front. Psychiatry* 6:32. 10.3389/fpsyt.2015.00032 25806005 PMC4353372

[B29] El Assar M. Angulo J. Carnicero J. A. Walter S. García-García F. J. López-Hernández E. (2017). Frailty is associated with lower expression of genes involved in cellular response to stress: results from the Toledo study for healthy aging. *J. Am. Med. Dir. Assoc.* 18 734.e1–734.e7. 10.1016/j.jamda.2017.04.019 28647579

[B30] Enoch M. A. Shen P. H. Ducci F. Yuan Q. Liu J. White K. V. (2008). Common genetic origins for EEG, alcoholism and anxiety: the role of CRH-BP. *PLoS One* 3:e3620. 10.1371/journal.pone.0003620 18974851 PMC2575401

[B31] Epel E. S. Blackburn E. H. Lin J. Dhabhar F. S. Adler N. E. Morrow J. D. (2004). Accelerated telomere shortening in response to life stress. *Proc. Natl. Acad. Sci. U.S.A.* 101 17312–17315. 10.1073/pnas.0407162101 15574496 PMC534658

[B32] Flory J. D. Yehuda R. (2015). Comorbidity between post-traumatic stress disorder and major depressive disorder: alternative explanations and treatment considerations. *Dialogues Clin. Neurosci.* 17 141–150. 10.31887/DCNS.2015.17.2/jflory 26246789 PMC4518698

[B33] Gray T. S. (1993). Amygdaloid CRF pathways. Role in autonomic, neuroendocrine, and behavioral responses to stress. *Ann. N. Y. Acad. Sci.* 697 53–60. 10.1111/j.1749-6632.1993.tb49922.x 8257022

[B34] Guex N. Peitsch M. C. Schwede T. (2009). Automated comparative protein structure modeling with SWISS-MODEL and Swiss-PdbViewer: a historical perspective. *Electrophoresis* 30(Suppl. 1) S162–S173. 10.1002/elps.200900140 19517507

[B35] Haass-Koffler C. L. (2018). The corticotropin releasing factor binding protein: a strange case of Dr. Jekyll and Mr. Hyde in the stress system? *Alcohol* 72 3–8. 10.1016/j.alcohol.2017.10.001 29510883 PMC5899053

[B36] Haass-Koffler C. L. Bartlett S. E. (2012). Stress and addiction: contribution of the corticotropin releasing factor (CRF) system in neuroplasticity. *Front. Mol. Neurosci.* 5:91. 10.3389/fnmol.2012.00091 22973190 PMC3434418

[B37] Haass-Koffler C. L. Henry A. T. Melkus G. Simms J. A. Naemmuddin M. Nielsen C. K. (2016). Defining the role of corticotropin releasing factor binding protein in alcohol consumption. *Transl. Psychiatry* 6:e953. 10.1038/tp.2016.208 27845775 PMC5314120

[B38] Haass-Koffler C. L. Naeemuddin M. Bartlett S. E. (2012). An analytical tool that quantifies cellular morphology changes from three-dimensional fluorescence images. *J. Vis. Exp.* 31:e4233. 10.3791/4233 22951512 PMC3486772

[B39] Haass-Koffler C. L. Swift R. M. Leggio L. (2018). Noradrenergic targets for the treatment of alcohol use disorder. *Psychopharmacology* 235 1625–1634. 10.1007/s00213-018-4843-6 29460163 PMC5995154

[B40] Hammen C. (2005). Stress and depression. *Annu. Rev. Clin. Psychol.* 1 293–319. 17716090 10.1146/annurev.clinpsy.1.102803.143938

[B41] Heinrichs S. C. Menzaghi F. Merlo Pich E. Britton K. T. Koob G. F. (1995). The role of CRF in behavioral aspects of stress. *Ann. N. Y. Acad. Sci.* 771 92–104. 10.1111/j.1749-6632.1995.tb44673.x 8597448

[B42] Hemmerle A. M. Herman J. P. Seroogy K. B. (2012). Stress, depression and Parkinson’s disease. *Exp. Neurol.* 233 79–86. 22001159 10.1016/j.expneurol.2011.09.035 PMC3268878

[B43] Herringa R. J. Nanda S. A. Hsu D. T. Roseboom P. H. Kalin N. H. (2004). The effects of acute stress on the regulation of central and basolateral amygdala CRF-binding protein gene expression. *Brain Res. Mol. Brain Res.* 131 17–25. 10.1016/j.molbrainres.2004.08.005 15530648

[B44] Jiang H. Ju Z. Rudolph K. L. (2007). Telomere shortening and ageing. *Z. Gerontol. Geriatr.* 40 314–324. 17943234 10.1007/s00391-007-0480-0

[B45] Karolyi I. J. Burrows H. L. Ramesh T. M. Nakajima M. Lesh J. S. Seong E. (1999). Altered anxiety and weight gain in corticotropin-releasing hormone-binding protein-deficient mice. *Proc. Natl. Acad. Sci. U.S.A.* 96 11595–11600. 10.1073/pnas.96.20.11595 10500222 PMC18079

[B46] Keller-Wood M. E. Dallman M. F. (1984). Corticosteroid inhibition of ACTH secretion. *Endocr. Rev.* 5 1–24. 10.1210/edrv-5-1-1 6323158

[B47] Kemp C. F. Woods R. J. Lowry P. J. (1998). The corticotrophin-releasing factor-binding protein: an act of several parts. *Peptides* 19 1119–1128. 10.1016/s0196-9781(98)00057-6 9700765

[B48] Kertes D. A. Kalsi G. Prescott C. A. Kuo P. H. Patterson D. G. Walsh D. (2011). Neurotransmitter and neuromodulator genes associated with a history of depressive symptoms in individuals with alcohol dependence. *Alcohol. Clin. Exp. Res.* 35 496–505. 10.1111/j.1530-0277.2010.01366.x 21143246 PMC3116055

[B49] Ketchesin K. D. Stinnett G. S. Seasholtz A. F. (2016). Binge drinking decreases corticotropin-releasing factor-binding protein expression in the medial prefrontal cortex of mice. *Alcohol. Clin. Exp. Res.* 40 1641–1650. 10.1111/acer.13119 27374820 PMC4961588

[B50] Ketchesin K. D. Stinnett G. S. Seasholtz A. F. (2017). Corticotropin-releasing hormone-binding protein and stress: from invertebrates to humans. *Stress* 20 449–464. 10.1080/10253890.2017.1322575 28436309 PMC7885796

[B51] Koob G. F. (2008). A role for brain stress systems in addiction. *Neuron* 59 11–34. 10.1016/j.neuron.2008.06.012 18614026 PMC2748830

[B52] Koob G. F. Le Moal M. (2008). Review. Neurobiological mechanisms for opponent motivational processes in addiction. *Philos. Trans. R. Soc. Lond. B Biol. Sci.* 363 3113–3123. 10.1098/rstb.2008.0094 18653439 PMC2607326

[B53] Koob G. Kreek M. J. (2007). Stress, dysregulation of drug reward pathways, and the transition to drug dependence. *Am. J. Psychiatry* 164 1149–1159. 10.1176/appi.ajp.2007.05030503 17671276 PMC2837343

[B54] Levran O. Peles E. Randesi M. Correa da Rosa J. Shen P. H. Rotrosen J. (2018). Genetic variations in genes of the stress response pathway are associated with prolonged abstinence from heroin. *Pharmacogenomics* 19 333–341. 10.2217/pgs-2017-0179 29465008 PMC5941712

[B55] Levran O. Randesi M. Li Y. Rotrosen J. Ott J. Adelson M. (2014). Drug addiction and stress-response genetic variability: association study in African Americans. *Ann. Hum. Genet.* 78 290–298. 10.1111/ahg.12064 24766650 PMC4065216

[B56] Li C. Zhao R. Gao K. Wei Z. Yin M. Y. Lau L. T. (2011). Astrocytes: implications for neuroinflammatory pathogenesis of Alzheimer’s disease. *Curr. Alzheimer Res.* 8 67–80. 10.2174/156720511794604543 21143158

[B57] Lijffijt M. Hu K. Swann A. C. (2014). Stress modulates illness-course of substance use disorders: a translational review. *Front. Psychiatry* 5:83. 10.3389/fpsyt.2014.00083 25101007 PMC4101973

[B58] Linton E. A. Wolfe C. D. Behan D. P. Lowry P. J. (1988). A specific carrier substance for human corticotrophin releasing factor in late gestational maternal plasma which could mask the ACTH-releasing activity. *Clin. Endocrinol.* 28 315–324. 10.1111/j.1365-2265.1988.tb01218.x 2844451

[B59] Locci A. Yan Y. Rodriguez G. Dong H. (2021). Sex differences in CRF1, CRF, and CRFBP expression in C57BL/6J mouse brain across the lifespan and in response to acute stress. *J. Neurochem.* 158 943–959. 10.1111/jnc.15157 32813270 PMC9811412

[B60] Lombardo K. A. Herringa R. J. Balachandran J. S. Hsu D. T. Bakshi V. P. Roseboom P. H. (2001). Effects of acute and repeated restraint stress on corticotropin-releasing hormone binding protein mRNA in rat amygdala and dorsal hippocampus. *Neurosci. Lett.* 302 81–84. 10.1016/s0304-3940(01)01680-9 11290392

[B61] Lovejoy D. A. Aubry J. M. Turnbull A. Sutton S. Potter E. Yehling J. (1998). Ectopic expression of the CRF-binding protein: minor impact on HPA axis regulation but induction of sexually dimorphic weight gain. *J. Neuroendocrinol.* 10 483–491. 10.1046/j.1365-2826.1998.00206.x 9700675

[B62] Lovinger D. M. (1997). Serotonin’s role in alcohol’s effects on the brain. *Alcohol Health Res. World* 21 114–120. 15704346 PMC6826824

[B63] Maciejewski D. Crowe P. D. De Souza E. B. Behan D. P. (1996). Regulation of corticotropin-releasing factor-binding protein expression in cultured rat astrocytes. *J. Pharmacol. Exp. Ther.* 278 455–461. 8768691

[B64] McCauley J. L. Killeen T. Gros D. F. Brady K. T. Back S. E. (2012). Posttraumatic stress disorder and co-occurring substance use disorders: advances in assessment and treatment. *Clin. Psychol.* 19 283–304. 10.1111/cpsp.12006 24179316 PMC3811127

[B65] McClennen S. J. Seasholtz A. F. (1999). Transcriptional regulation of corticotropin-releasing hormone-binding protein gene expression in astrocyte cultures. *Endocrinology* 140 4095–4103. 10.1210/endo.140.9.6978 10465281

[B66] McClennen S. J. Cortright D. N. Seasholtz A. F. (1998). Regulation of pituitary corticotropin-releasing hormone-binding protein messenger ribonucleic acid levels by restraint stress and adrenalectomy. *Endocrinology* 139 4435–4441. 10.1210/endo.139.11.6311 9794449

[B67] Miller D. B. O’Callaghan J. P. (2008). Do early-life insults contribute to the late-life development of Parkinson and Alzheimer diseases? *Metabolism* 57(Suppl. 2) S44–S49. 10.1016/j.metabol.2008.07.011 18803966

[B68] Mosher K. I. Wyss-Coray T. (2014). Microglial dysfunction in brain aging and Alzheimer’s disease. *Biochem. Pharmacol.* 88 594–604. 10.1016/j.bcp.2014.01.008 24445162 PMC3972294

[B69] Mukhara D. Banks M. L. Neigh G. N. (2018). Stress as a risk factor for substance use disorders: a mini-review of molecular mediators. *Front. Behav. Neurosci.* 12:309. 10.3389/fnbeh.2018.00309 30622460 PMC6308626

[B70] Nobili A. Latagliata E. C. Viscomi M. T. Cavallucci V. Cutuli D. Giacovazzo G. (2017). Dopamine neuronal loss contributes to memory and reward dysfunction in a model of Alzheimer’s disease. *Nat. Commun.* 8:14727. 10.1038/ncomms14727 28367951 PMC5382255

[B71] Nolte T. Guiney J. Fonagy P. Mayes L. C. Luyten P. (2011). Interpersonal stress regulation and the development of anxiety disorders: an attachment-based developmental framework. *Front. Behav. Neurosci.* 5:55. 10.3389/fnbeh.2011.00055 21960962 PMC3177081

[B72] O’Connell C. P. Goldstein-Piekarski A. N. Nemeroff C. B. Schatzberg A. F. Debattista C. Carrillo-Roa T. (2018). Antidepressant outcomes predicted by genetic variation in corticotropin-releasing hormone binding protein. *Am. J. Psychiatry* 175 251–261. 10.1176/appi.ajp.2017.17020172 29241359 PMC5832545

[B73] Overton P. G. Clark D. (1997). Burst firing in midbrain dopaminergic neurons. *Brain Res. Brain Res. Rev.* 25 312–334. 10.1016/s0165-0173(97)00039-8 9495561

[B74] Peavy G. M. Jacobson M. W. Salmon D. P. Gamst A. C. Patterson T. L. Goldman S. (2012). The influence of chronic stress on dementia-related diagnostic change in older adults. *Alzheimer Dis. Assoc. Disord.* 26 260–266. 10.1097/WAD.0b013e3182389a9c 22037597 PMC3290680

[B75] Pêgo J. M. Sousa J. C. Almeida O. F. Sousa N. (2010). Stress and the neuroendocrinology of anxiety disorders. *Curr. Top. Behav. Neurosci.* 2 97–117. 10.1007/7854_2009_13 21309108

[B76] Peles E. Levran O. Randesi M. Ott J. Kreek M. J. Adelson M. (2019). Genetic variant in the CRH-binding protein gene (CRHBP) is associated with cessation of cocaine use in methadone maintenance patients with opioid addiction. *J. Addict. Med.* 13 430–435. 10.1097/ADM.0000000000000515 30844877

[B77] Petraglia F. Potter E. Cameron V. A. Sutton S. Behan D. P. Woods R. J. (1993). Corticotropin-releasing factor-binding protein is produced by human placenta and intrauterine tissues. *J. Clin. Endocrinol. Metab.* 77 919–924. 10.1210/jc.77.4.919 8408466

[B78] Phatnani H. Maniatis T. (2015). Astrocytes in neurodegenerative disease. *Cold Spring Harb. Perspect. Biol.* 7:a020628. 10.1101/cshperspect.a020628 25877220 PMC4448607

[B79] Pisarska M. Mulchahey J. J. Welge J. A. Geracioti T. D. Jr. Kasckow J. W. (2000). Age-related alterations in emotional behaviors and amygdalar corticotropin-releasing factor (CRF) and CRF-binding protein expression in aged Fischer 344 rats. *Brain Res.* 877 184–190. 10.1016/s0006-8993(00)02606-8 10986331

[B80] Pomara N. Greenberg W. M. Branford M. D. Doraiswamy P. M. (2003). Therapeutic implications of HPA axis abnormalities in Alzheimer’s disease: review and update. *Psychopharmacol. Bull.* 37 120–134. 14674372

[B81] Potter E. Behan D. P. Fischer W. H. Linton E. A. Lowry P. J. Vale W. W. (1991). Cloning and characterization of the cDNAs for human and rat corticotropin releasing factor-binding proteins. *Nature* 349 423–426. 10.1038/349423a0 1846945

[B82] Potter E. Behan D. P. Linton E. A. Lowry P. J. Sawchenko P. E. Vale W. W. (1992). The central distribution of a corticotropin-releasing factor (CRF)-binding protein predicts multiple sites and modes of interaction with CRF. *Proc. Natl. Acad. Sci. U.S.A.* 89 4192–4196. 10.1073/pnas.89.9.4192 1315056 PMC525659

[B83] Raff H. (1993). Interactions between neurohypophysial hormones and the ACTH-adrenocortical axis. *Ann. N. Y. Acad. Sci.* 689 411–425. 10.1111/j.1749-6632.1993.tb55564.x 8396873

[B84] Ray L. A. (2011). Stress-induced and cue-induced craving for alcohol in heavy drinkers: preliminary evidence of genetic moderation by the OPRM1 and CRH-BP genes. *Alcohol. Clin. Exp. Res.* 35 166–174. 10.1111/j.1530-0277.2010.01333.x 21039637

[B85] Rea I. M. Gibson D. S. McGilligan V. McNerlan S. E. Alexander H. D. Ross O. A. (2018). Age and age-related diseases: role of inflammation triggers and cytokines. *Front. Immunol.* 9:586. 10.3389/fimmu.2018.00586 29686666 PMC5900450

[B86] Roberto M. Gilpin N. W. Siggins G. R. (2012). The central amygdala and alcohol: role of γ-aminobutyric acid, glutamate, and neuropeptides. *Cold Spring Harb. Perspect. Med.* 2:a012195. 10.1101/cshperspect.a012195 23085848 PMC3543070

[B87] Rodriguez F. D. Coveñas R. (2017). Targeting NPY, CRF/UCNs and NPS neuropeptide systems to treat alcohol use disorder (AUD). *Curr. Med. Chem.* 24 2528–2558. 10.2174/0929867324666170316120836 28302012

[B88] Roseboom P. H. Nanda S. A. Bakshi V. P. Trentani A. Newman S. M. Kalin N. H. (2007). Predator threat induces behavioral inhibition, pituitary-adrenal activation and changes in amygdala CRF-binding protein gene expression. *Psychoneuroendocrinology* 32 44–55. 10.1016/j.psyneuen.2006.10.002 17116372 PMC1847640

[B89] Roy A. Hodgkinson C. A. Deluca V. Goldman D. Enoch M. A. (2012). Two HPA axis genes, CRHBP and FKBP5, interact with childhood trauma to increase the risk for suicidal behavior. *J. Psychiatr. Res.* 46 72–79. 10.1016/j.jpsychires.2011.09.009 21978546 PMC3506169

[B90] Salim S. (2016). Oxidative stress: a potential link between emotional wellbeing and immune response. *Curr. Opin. Pharmacol.* 29 70–76. 27400336 10.1016/j.coph.2016.06.006

[B91] Sanders J. Nemeroff C. (2016). The CRF system as a therapeutic target for neuropsychiatric disorders. *Trends Pharmacol. Sci.* 37 1045–1054. 10.1016/j.tips.2016.09.004 27717506 PMC5121012

[B92] Sarnyai Z. Shaham Y. Heinrichs S. C. (2001). The role of corticotropin-releasing factor in drug addiction. *Pharmacol. Rev.* 53 209–243. 11356984

[B93] Schreiber A. L. Gilpin N. W. (2018). Corticotropin-releasing factor (CRF) neurocircuitry and neuropharmacology in alcohol drinking. *Handb. Exp. Pharmacol.* 248 435–471. 10.1007/164_2017_86 29374836 PMC6064393

[B94] Shaham Y. Erb S. Stewart J. (2000). Stress-induced relapse to heroin and cocaine seeking in rats: a review. *Brain Res. Brain Res. Rev.* 33 13–33. 10.1016/s0165-0173(00)00024-2 10967352

[B95] Simms J. A. Haass-Koffler C. L. Bito-Onon J. Li R. Bartlett S. E. (2012). Mifepristone in the central nucleus of the amygdala reduces yohimbine stress-induced reinstatement of ethanol-seeking. *Neuropsychopharmacology* 37 906–918. 10.1038/npp.2011.268 22048462 PMC3280651

[B96] Sinha R. (2008). Chronic stress, drug use, and vulnerability to addiction. *Ann. N. Y. Acad. Sci.* 1141 105–130. 18991954 10.1196/annals.1441.030 PMC2732004

[B97] Slater P. G. Cerda C. A. Pereira L. A. Andrés M. E. Gysling K. (2016). CRF binding protein facilitates the presence of CRF type 2α receptor on the cell surface. *Proc. Natl. Acad. Sci. U.S.A.* 113 4075–4080. 10.1073/pnas.1523745113 27035969 PMC4839449

[B98] Speert D. B. Sj M. C. Seasholtz A. F. (2002). Sexually dimorphic expression of corticotropin-releasing hormone-binding protein in the mouse pituitary. *Endocrinology* 143 4730–4741. 12446601 10.1210/en.2002-220556

[B99] Spierling S. R. Zorrilla E. P. (2017). Don’t stress about CRF: assessing the translational failures of CRF(1)antagonists. *Psychopharmacology* 234 1467–1481. 10.1007/s00213-017-4556-2 28265716 PMC5420464

[B100] Stinnett G. S. Westphal N. J. Seasholtz A. F. (2015). Pituitary CRH-binding protein and stress in female mice. *Physiol. Behav.* 150 16–23. 25731977 10.1016/j.physbeh.2015.02.050 PMC4546865

[B101] Su H. Wang Z. Zhao M. (2018). Association between stress pathway gene (CRHR1\CRHBP) polymorphisms and heroin dependence. *J. Clin. Neurosci.* 54 33–38. 10.1016/j.jocn.2018.05.009 29853227

[B102] Sutton S. W. Behan D. P. Lahrichi S. L. Kaiser R. Corrigan A. Lowry P. (1995). Ligand requirements of the human corticotropin-releasing factor-binding protein. *Endocrinology* 136 1097–1102. 10.1210/endo.136.3.7867564 7867564

[B103] Swaab D. F. Dubelaar E. J. Hofman M. A. Scherder E. J. van Someren E. J. Verwer R. W. (2002). Brain aging and Alzheimer’s disease; use it or lose it. *Prog. Brain Res.* 138 343–373. 12432778 10.1016/S0079-6123(02)38086-5

[B104] Swerdlow R. H. (2011). Brain aging, Alzheimer’s disease, and mitochondria. *Biochim. Biophys. Acta* 1812 1630–1639. 21920438 10.1016/j.bbadis.2011.08.012 PMC3210037

[B105] Tartter M. A. Ray L. A. (2012). A prospective study of stress and alcohol craving in heavy drinkers. *Pharmacol. Biochem. Behav.* 101 625–631. 10.1016/j.pbb.2012.03.007 22446386

[B106] Tasker J. G. Herman J. P. (2011). Mechanisms of rapid glucocorticoid feedback inhibition of the hypothalamic-pituitary-adrenal axis. *Stress* 14 398–406. 10.3109/10253890.2011.586446 21663538 PMC4675656

[B107] Thase M. E. (2008). Are SNRIs more effective than SSRIs? A review of the current state of the controversy. *Psychopharmacol. Bull.* 41 58–85. 18668017

[B108] Trantham-Davidson H. Chandler L. J. (2015). Alcohol-induced alterations in dopamine modulation of prefrontal activity. *Alcohol* 49 773–779. 10.1016/j.alcohol.2015.09.001 26558348 PMC4691370

[B109] Ungless M. A. Singh V. Crowder T. L. Yaka R. Ron D. Bonci A. (2003). Corticotropin-releasing factor requires CRF binding protein to potentiate NMDA receptors *via* CRF receptor 2 in dopamine neurons. *Neuron* 39 401–407. 10.1016/s0896-6273(03)00461-6 12895416

[B110] Van Den Eede F. Venken T. Del-Favero J. Norrback K. F. Souery D. Nilsson L. G. (2007). Single nucleotide polymorphism analysis of corticotropin-releasing factor-binding protein gene in recurrent major depressive disorder. *Psychiatry Res.* 153 17–25. 17599466 10.1016/j.psychres.2006.12.018

[B111] Vandael D. Gounko N. V. (2019). Corticotropin releasing factor-binding protein (CRF-BP) as a potential new therapeutic target in Alzheimer’s disease and stress disorders. *Transl. Psychiatry* 9:272. 10.1038/s41398-019-0581-8 31641098 PMC6805916

[B112] Wang B. You Z. B. Rice K. C. Wise R. A. (2007). Stress-induced relapse to cocaine seeking: roles for the CRF(2) receptor and CRF-binding protein in the ventral tegmental area of the rat. *Psychopharmacology* 193 283–294. 17437087 10.1007/s00213-007-0782-3

[B113] Wang H. L. Morales M. (2008). Corticotropin-releasing factor binding protein within the ventral tegmental area is expressed in a subset of dopaminergic neurons. *J. Comp. Neurol.* 509 302–318. 10.1002/cne.21751 18478589 PMC2575090

[B114] Waterhouse A. Bertoni M. Bienert S. Studer G. Tauriello G. Gumienny R. (2018). SWISS-MODEL: homology modelling of protein structures and complexes. *Nucleic Acids Res.* 46 W296–W303. 29788355 10.1093/nar/gky427 PMC6030848

[B115] Wiersielis K. R. Ceretti A. Hall A. Famularo S. T. Salvatore M. Ellis A. S. (2019). Sex differences in corticotropin releasing factor regulation of medial septum-mediated memory formation. *Neurobiol. Stress* 10:100150. 10.1016/j.ynstr.2019.100150 30937355 PMC6430617

[B116] Wolf E. J. Morrison F. G. (2017). Traumatic stress and accelerated cellular aging: from epigenetics to cardiometabolic disease. *Curr. Psychiatry Rep.* 19:75. 10.1007/s11920-017-0823-5 28852965 PMC5588711

[B117] Woods R. J. Kemp C. F. David J. Sumner I. G. Lowry P. J. (1999). Cleavage of recombinant human corticotropin-releasing factor (CRF)-binding protein produces a 27-kilodalton fragment capable of binding CRF. *J. Clin. Endocrinol. Metab.* 84 2788–2794. 10.1210/jcem.84.8.5898 10443681

[B118] Yegorov Y. E. Poznyak A. V. Nikiforov N. G. Sobenin I. A. Orekhov A. N. (2020). The link between chronic stress and accelerated aging. *Biomedicines* 8:198. 10.3390/biomedicines8070198 PMC7400286 32645916

[B119] You C. Vandegrift B. Brodie M. S. (2018). Ethanol actions on the ventral tegmental area: novel potential targets on reward pathway neurons. *Psychopharmacology* 235 1711–1726. 10.1007/s00213-018-4875-y 29549390 PMC5949141

